# Supplementary material for: A semi-supervised approach uncovers thousands of intragenic enhancers differentially activated in human cells
Source: BMC Genomics. 2015 Jul 14;16(1):523. doi: 10.1186/s12864-015-1704-0 (PMC4501197; doi:10.1186/s12864-015-1704-0)
Supplement: Additional file 1: — Contains the supplementary figures (S1-S19) and tables (S1-S5) cited in the text. [file 12864_2015_1704_MOESM1_ESM.pdf]

Supplementary Material

A semi-supervised approach uncovers thousands of intragenic enhancers differentially activated in human cells

*Juan González-Vallinas[1], Amadis Pagés[1], Babita Singh[1], Eduardo Eyras[1,2]**

[1]Universitat Pompeu Fabra, Dr Aiguader 88, E08003 Barcelona, Spain
[2]Catalan Institution for Research and Advanced Studies (ICREA), Passeig Lluís Companys 23, E08010 Barcelona, Spain

* Corresponding author: eduardo.eyras@upf.edu

Supplementary Figure 1

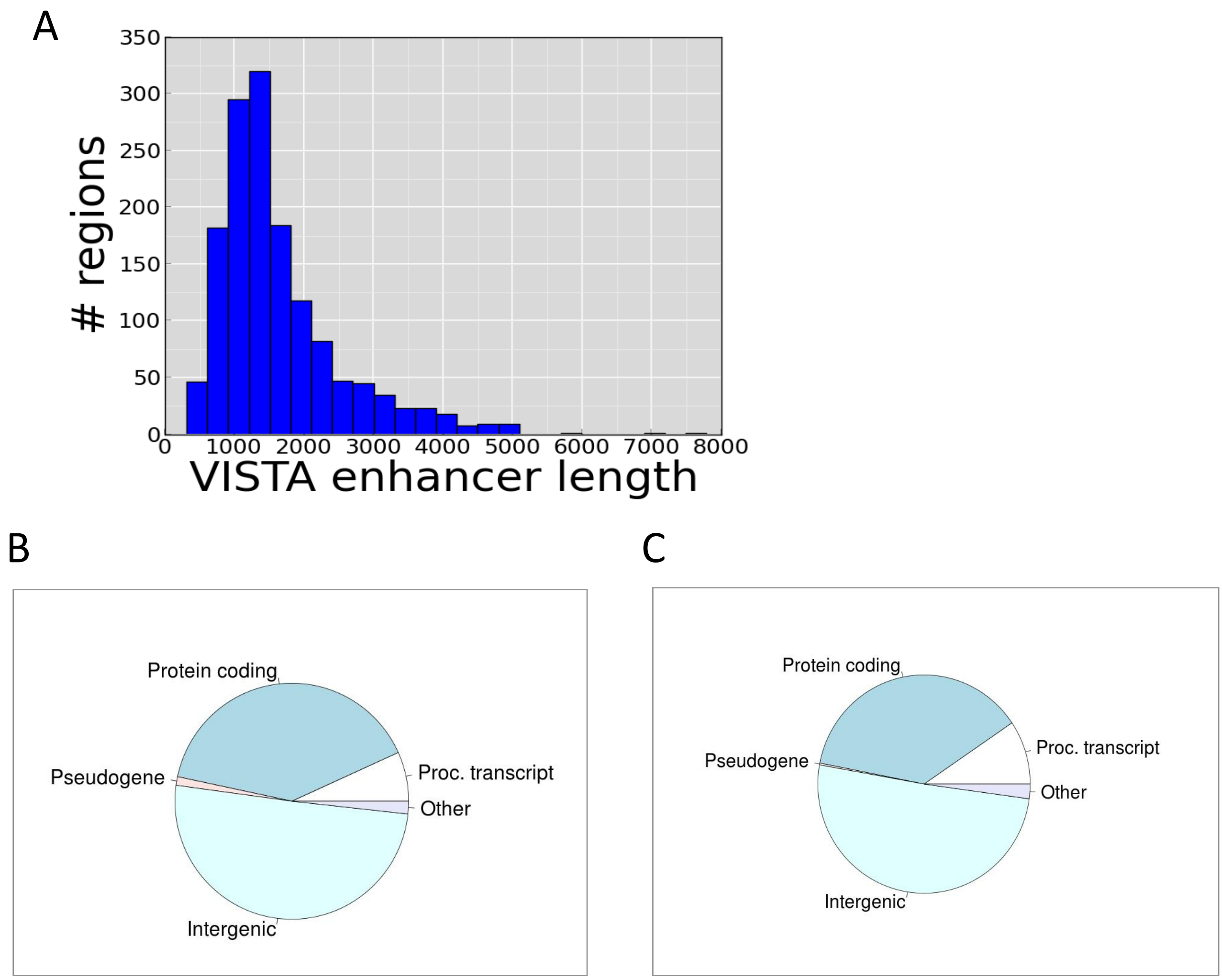

Supplementary Figure 1. Properties of the VISTA enhancers. A - Length distribution of human VISTA enhancers. The average length of the VISTA regions is 1637.9 nt (median 1383nt), standard deviation 891nt. Out of the 1447 experimentally validated regions, only 6 (0.41%) are above 5000 nucleotides. **B** - GENCODE coverage of the genome. Pie chart representing the percentage of bases covered by different annotated elements in the human genome (GENCODE V7): protein coding genes, Processed Transcripts, Pseudogenes, regions and Other. The label Other summarizes Immunoglobulin (Ig) variable chain and T-cell receptor (TcR) genes (active and silent), several types of small non-coding RNA and lincRNA. The rest of non annotated elements by GENCODE are classified as intergenic. **C** - VISTA elements positioning. Percentage of the VISTA regions that fall in one of the categories described above.

Supplementary Figure 2

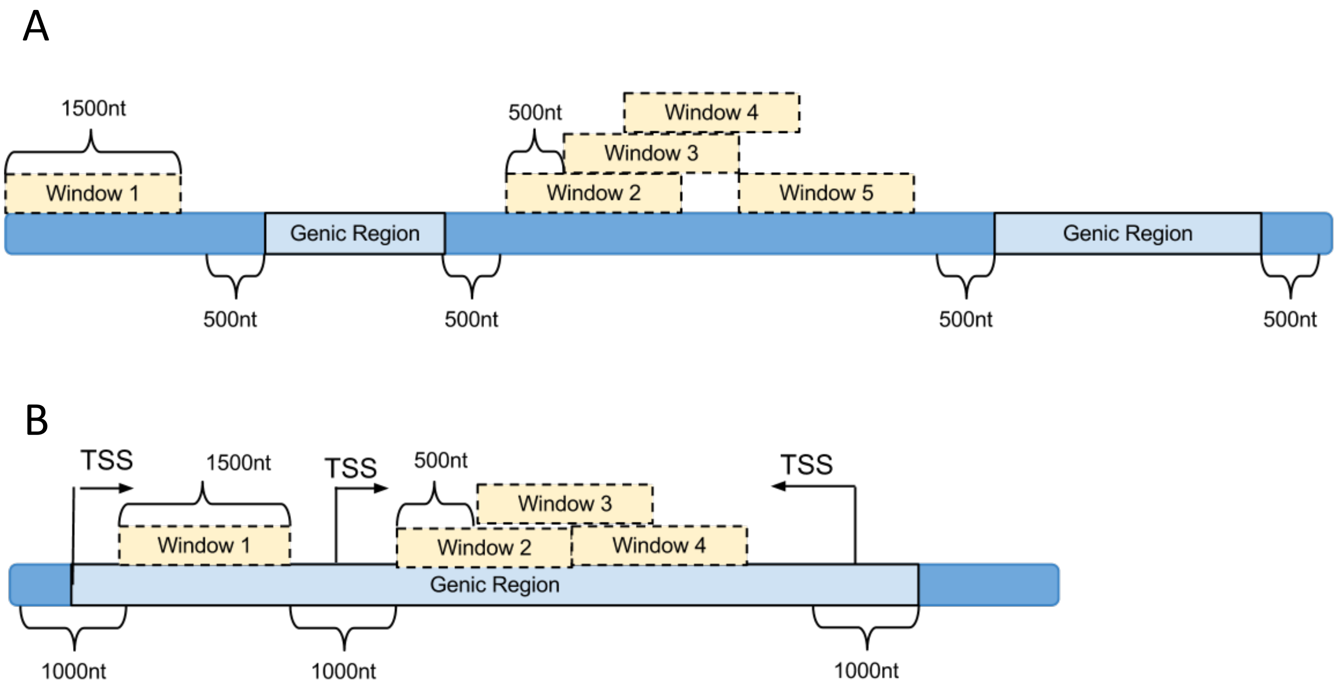

Supplementary Figure 2. Intergenic and Intragenic exploratory windows. A - The intergenic exploratory intergenic windows have a size of 1500nt and are overlapping 500nt from each other. In order to avoid mixing with promoter signal, they are at least 500nt away from Genic regions. **B -** Intragenic windows have the same size as the intergenic windows and are at least 500nt away from any TSS (either first or intragenic).

Supplementary Figure 3

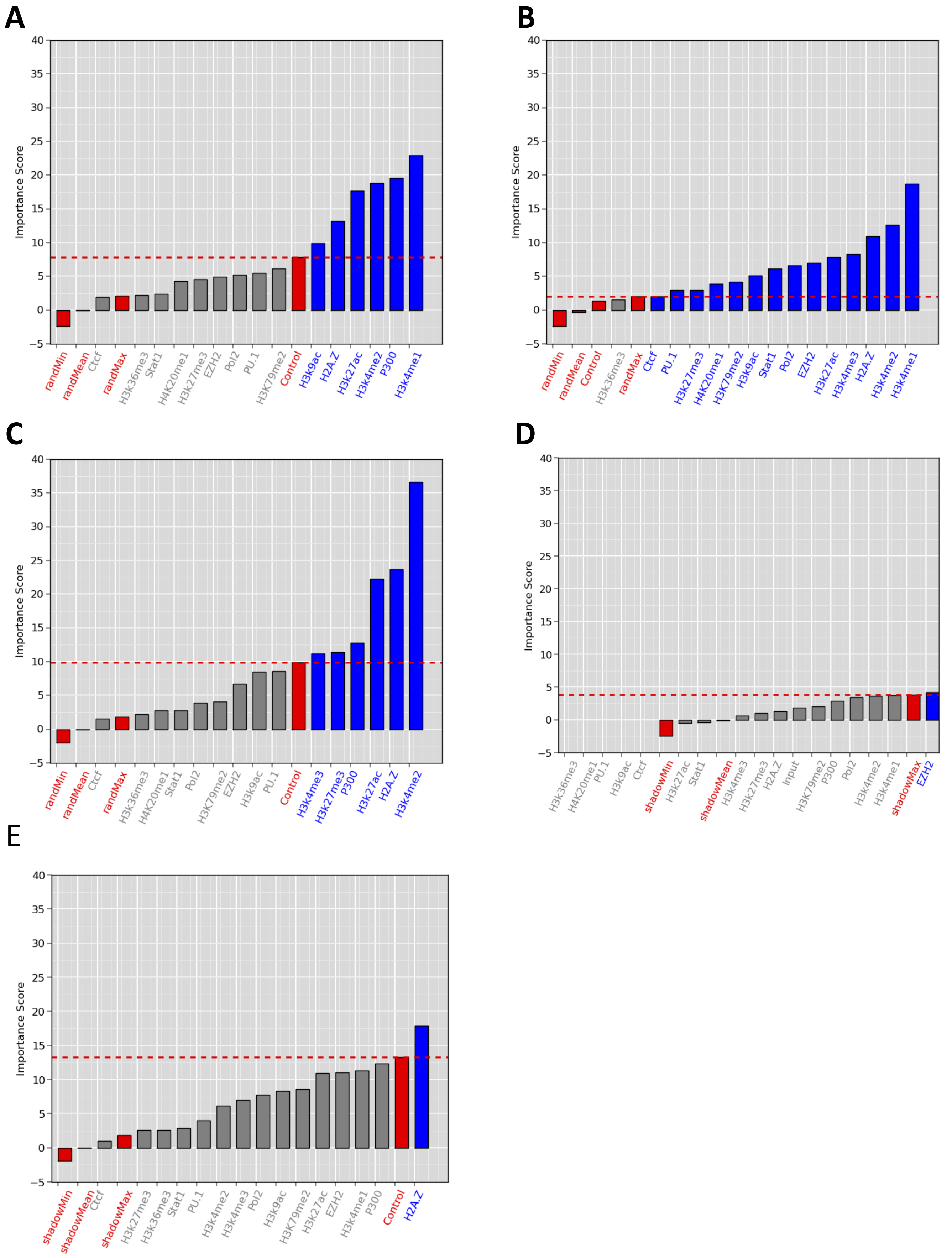

Supplementary Figure 3

Supplementary Figure 3. Feature Selection. A - Feature selection using H3K27ac as a correlation class. The bars represent the average importance score per feature after averaging over 10 random samples of 5000 intergenic windows extracting from all intergenic windows with non-zero signal in at least one cell type. Red labels and bars indicate the minimum (randMin), mean (randMean) and maximum (randMax) of the simulated replicates, as well as the ChIP-Seq with a non-specific antibody (Control). The red dashed line separates the relevant features (in blue) from the non-relevant features (in grey). **B** - Feature selection average scores using P300 as the correlation class. **C -** Feature selection average scores using H3K4me1 as the correlation class. **D -** Feature selection average scores using Control as the correlation class. The Control (ChIP-Seq experiment with no specific antibody) did not correlate significantly with any of the other features. On the other hand, **E -** Feature selection average scores using H4k20me1 as the correlation class. H4K20me1, which has been associated to transcription repression and heterochromatin (Balakrishnan et al. 2010, Beck et al. 2012) but not to enhancer activity, shows some correlation with H2A.Z, but no correlation with any other signal. Feature selection was performed using Boruta.

Supplementary Figure 4

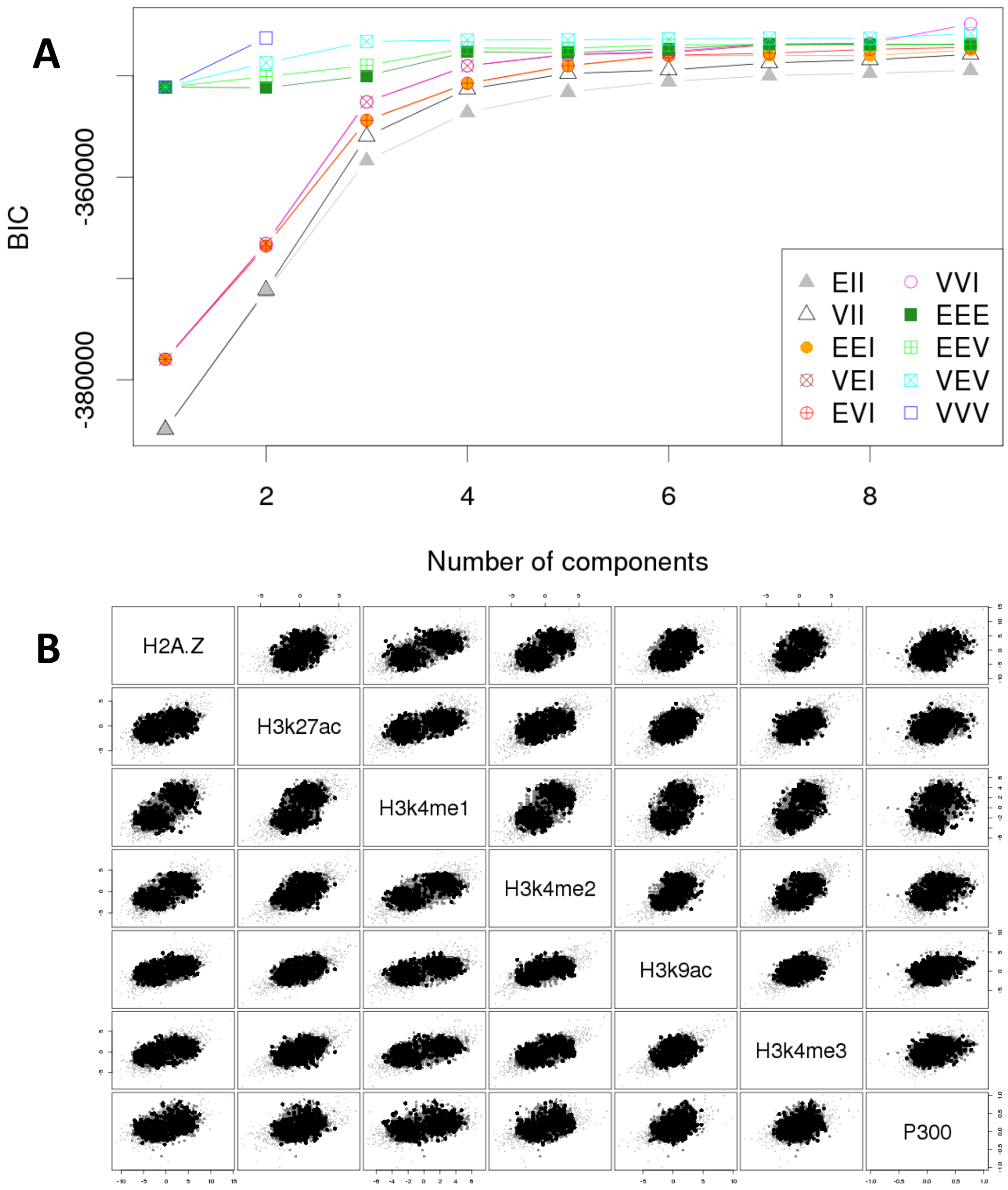

Supplementary Figure 4. Intergenic Clustering. A. The Bayesian Information Criterion (BIC) calculation with Mclust. The X axis represents the number of clusters, the Y axis the BIC score. Every line correspond to a different type of model. The model that scores higher and plateaus faster is VEV (Variable Volume, Equal Shape, Variable Orientation). **B.** Mclust visual representation of the uncertainty. Dark dots represent more uncertainty (less probable to be good predictions), lighter dots are more certain (more probable to be good predictions). As expected, bigger differences between K562 and GM12878 levels correlate with less uncertainty.

Supplementary Figure 5

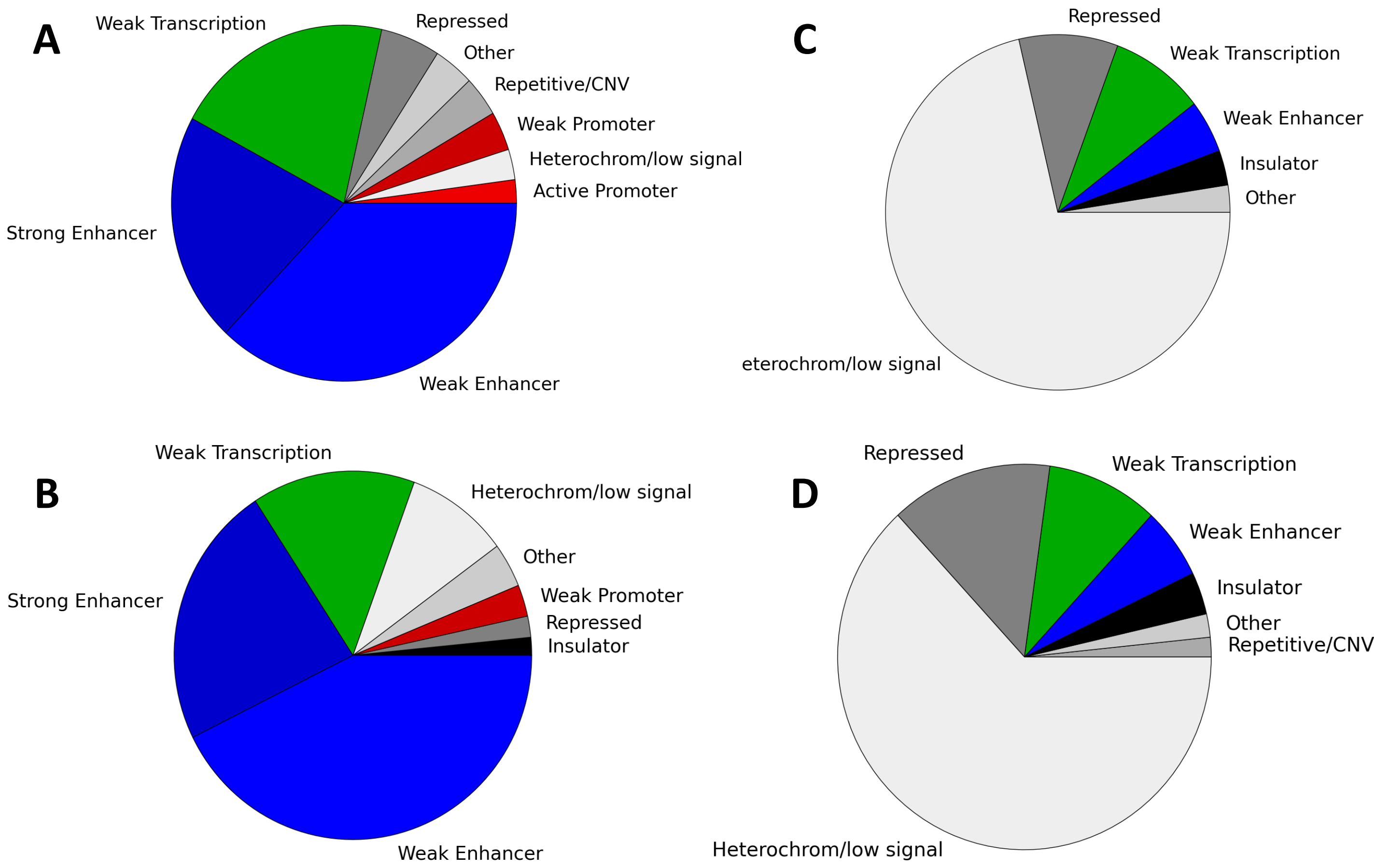

Supplementary Figure 5. Comparing intergenic predictions with ChromHMM predictions. A. Pie chart showing the overlap of our intergenic windows predicted to be active in K562 with different ChromHMM (Ernst et al. 2011) classes for K562. Our predictions overlap with ChromHMM weak (light blue) or strong (dark blue) enhancers, weak transcription (green), weak (dark read) and active (light red) candidate promoters, repressed regions (dark grey), repetitive regions (middle grey) and others (light grey). **B.** As in A, pie chart showing the overlap of enhancers predicted to be active in GM12878 with the ChromHMM classes for GM12878. **C.** Pie chart showing the overlap of our windows predicted as active in K562 (silent in GM12878) with ChromHMM classes for GM12878. The majority of windows overlap with heterochromatin regions (light grey) and repressed regions (dark grey). There are also some overlaps with weak enhancers (blue) and weak transcription (green). **D.** As per C., Pie chart showing the overlap of our windows predicted as active intergenic enhancers in GM12878 (silent in K562) with ChromHMM classes for K562. The majority of windows overlap with heterochromatin regions (light grey) and repressed regions (dark grey). There are also some overlaps with weak enhancers (blue) and weak transcription (green).

Supplementary Figure 5 (continued)

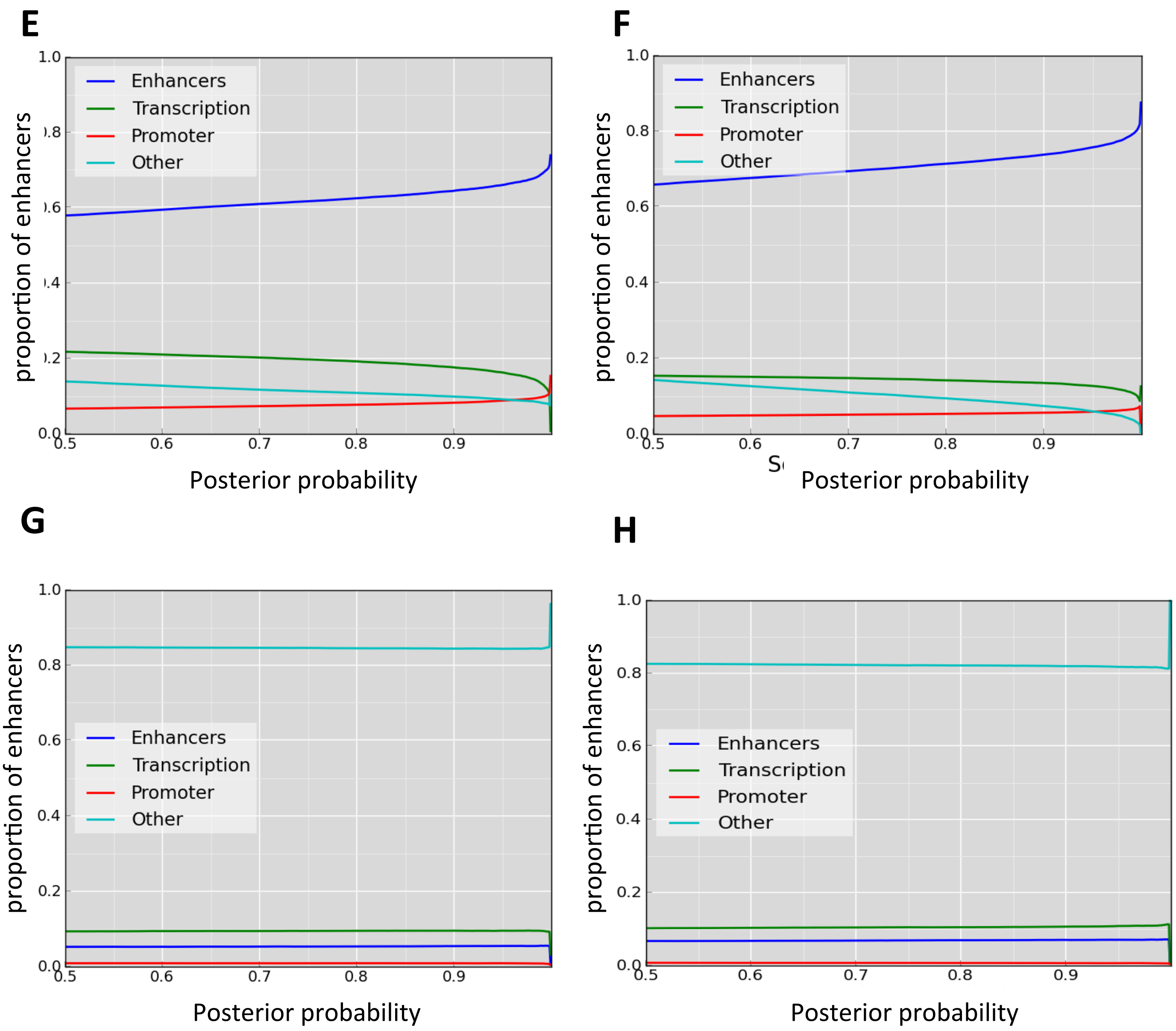

Supplementary Figure 5. Proportion of predicted enhancers that are labeled by ChromHMM in each cell line (y axis) as a function of the posterior probability (x axi), for active enhancers in K562 **(E)** and GM12878 **(F)** and of silent enhancers in K562 **(G)** and GM12878 **(H).** The lines indicate the proportion of overlap ChromHMM regions labelled as weak or strong enhancers (blue), transcription related classes (green), promoters (red) and repressed (repressed, heterochromatin and repetitive) (cyan) regions

Supplementary Figure 6

A

B

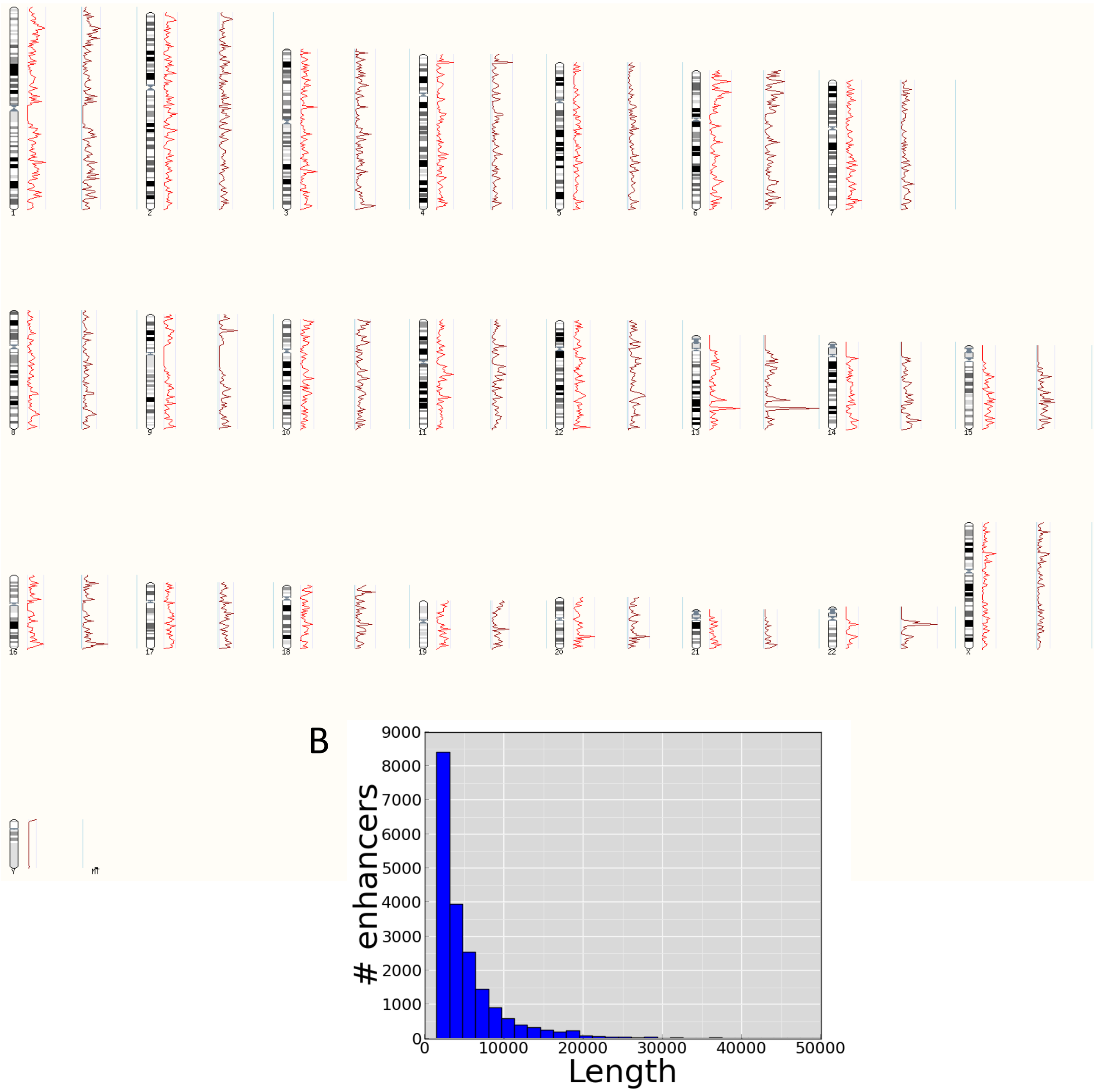

Supplementary Figure 6. A. Genome wide distribution of intergenic enhancers. Profile of the density of predicted intergenic enhancers (active and silent) along the human karyotype, for all predicted enhancers (dark red) and for enhancers of lengths < 5kb (light red). **B -** Length distribution of our predicted intergenic enhancers

Supplementary Figure 7

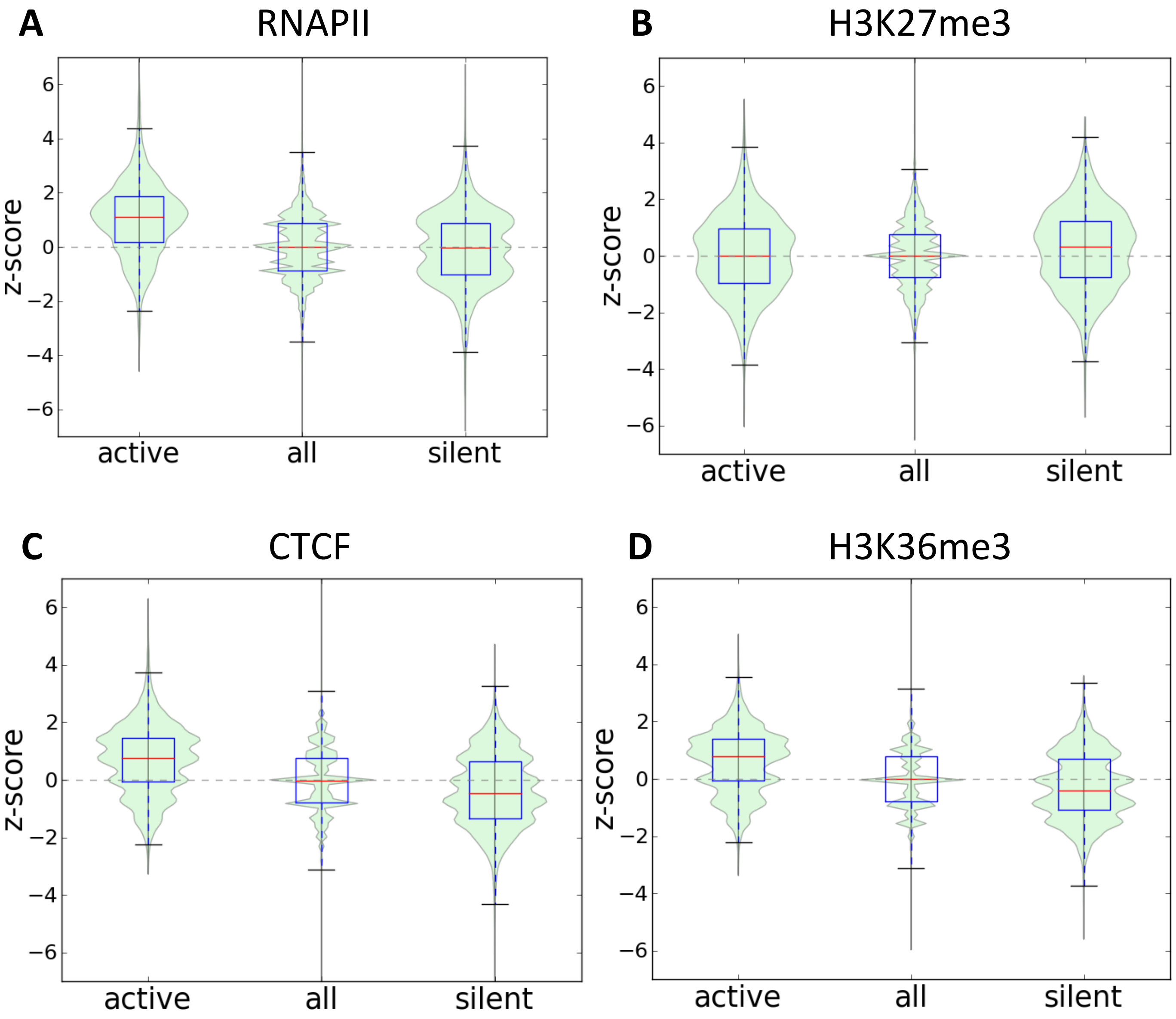

Supplementary Figure 7. Relative enrichment of RNAPII **(A)**, H3K27me3 **(B)**, CTCF **(C)** and H3K36me3 **(D)** in intergenic enhancers. Z-score distributions for our putative active and silent enhancers, as well as for all regions

Supplementary Figure 8

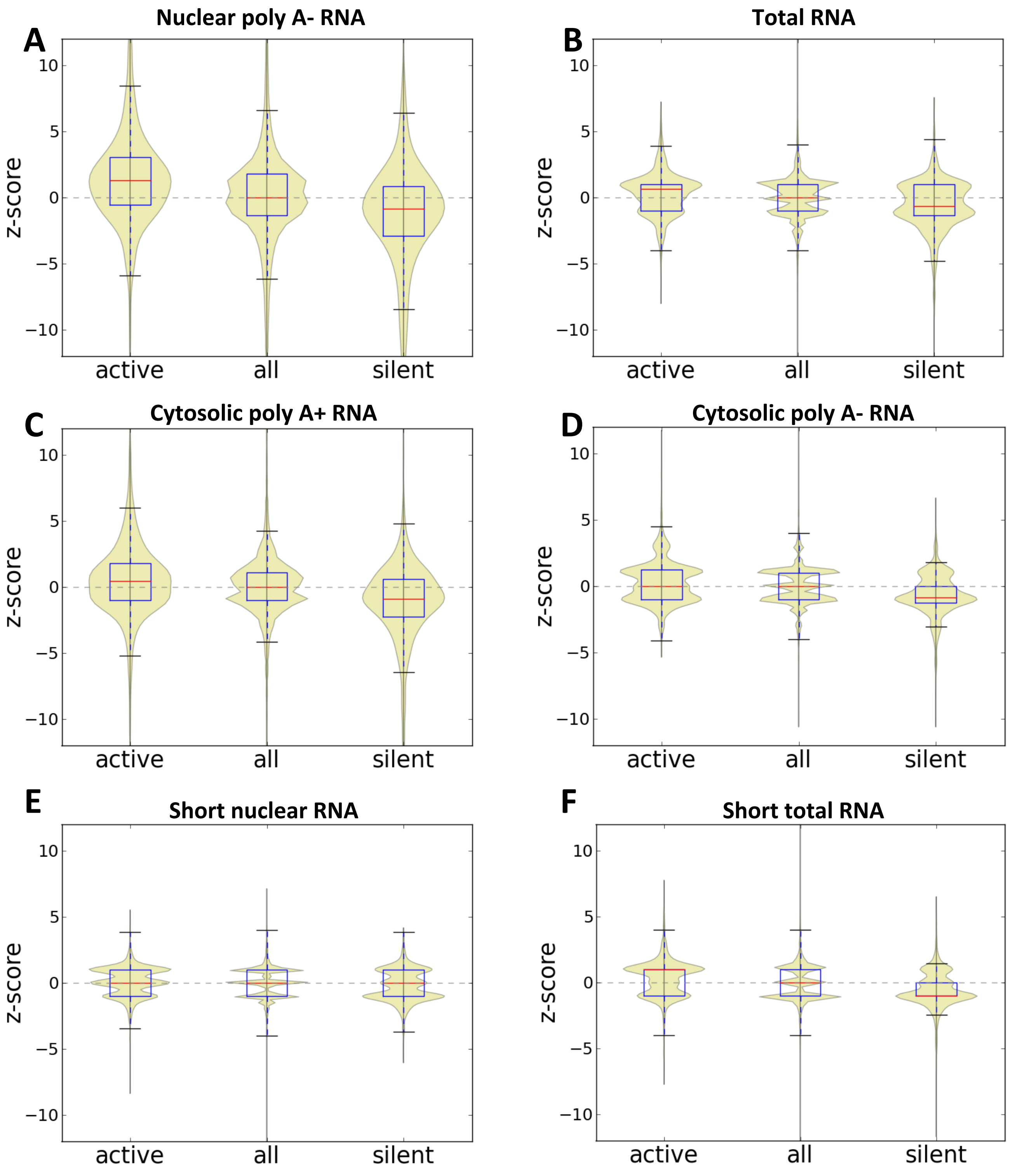

Supplementary Figure 8. Violin plots for the relative enrichment in K562 relative GM12878 in active and silent enhancers, as well as the distribution of all z-scores, for **(A)** nuclear long (>200nt) poly A- RNAs, **(B)** total RNA, **(C)** Cytosolic polyA+ RNAs, **(D)** Cytosolic polyA- RNAs, **(E)** Short (<200nt) nuclear RNAs and **(F)** Short (<200nt) Total RNAs.

Supplementary Figure 9

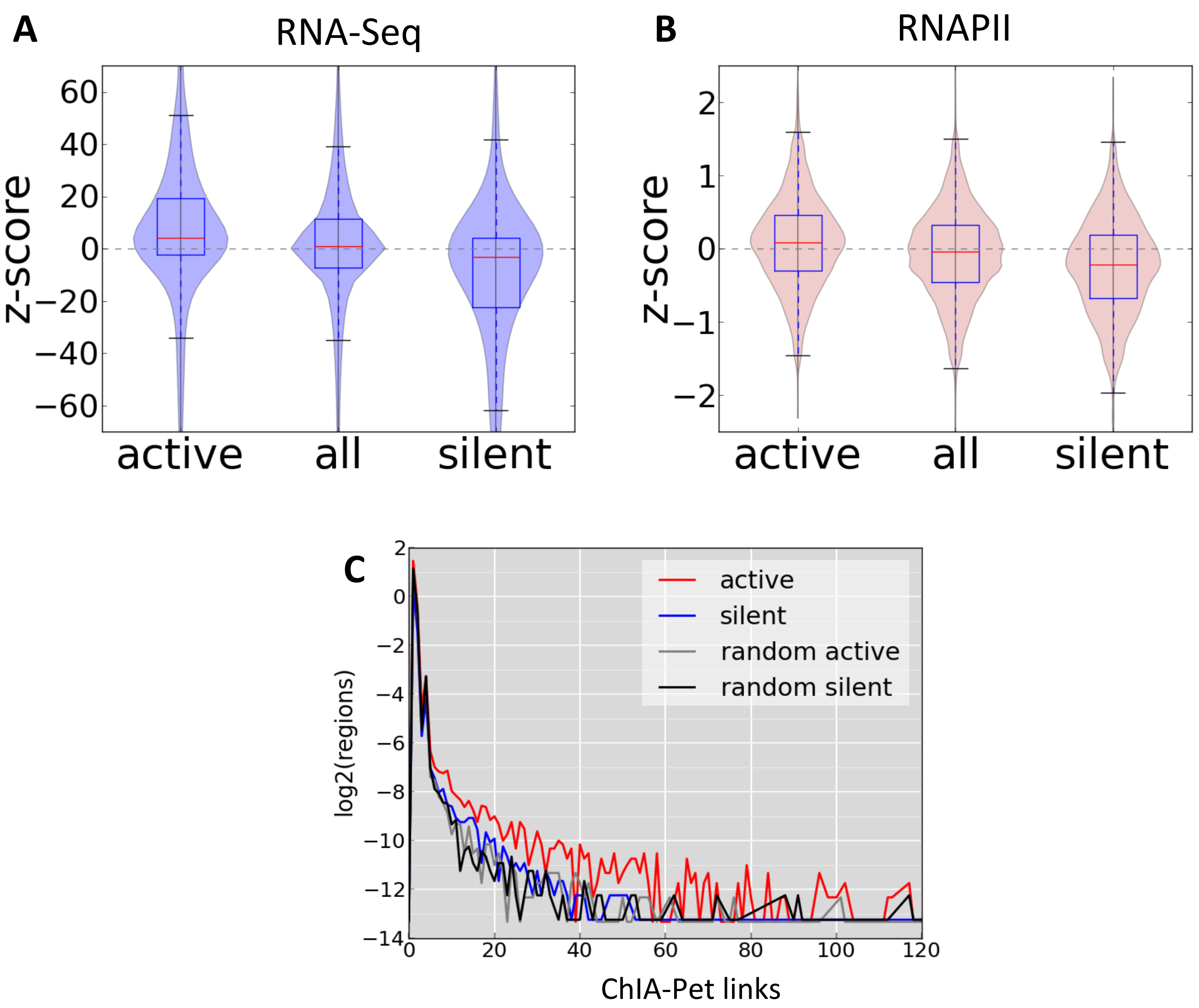

Supplementary Figure 9. Relative change in **(A)** gene expression and in **(B)** RNAPII at the TSS for genes. The Y axis measures the relative expression change in terms of a Z-score for genes associated to active (left violin plot) and silent enhancers (right violin plot), and for all genes (middle violin plots). The relative change in RNAPII density is measured in a 1kb window around the TSS. Genes were associated to the nearest predicted enhancer within a range of 10kb to 100kb from the TSS on either direction. **C -** Percentage of intergenic predicted enhancers linked by ChIA-PET to a nearby TSS**.** In the Y-axis we plot in log2-scale the fraction of regions with ChIA-PET links to a nearby TSS, for activated, silenced, random activated and random silenced enhancers. TSS – enhancer pairs are considered when all elements are located at least 3 kilobases away and as far as 100 kilobases.

Supplementary Figure 10

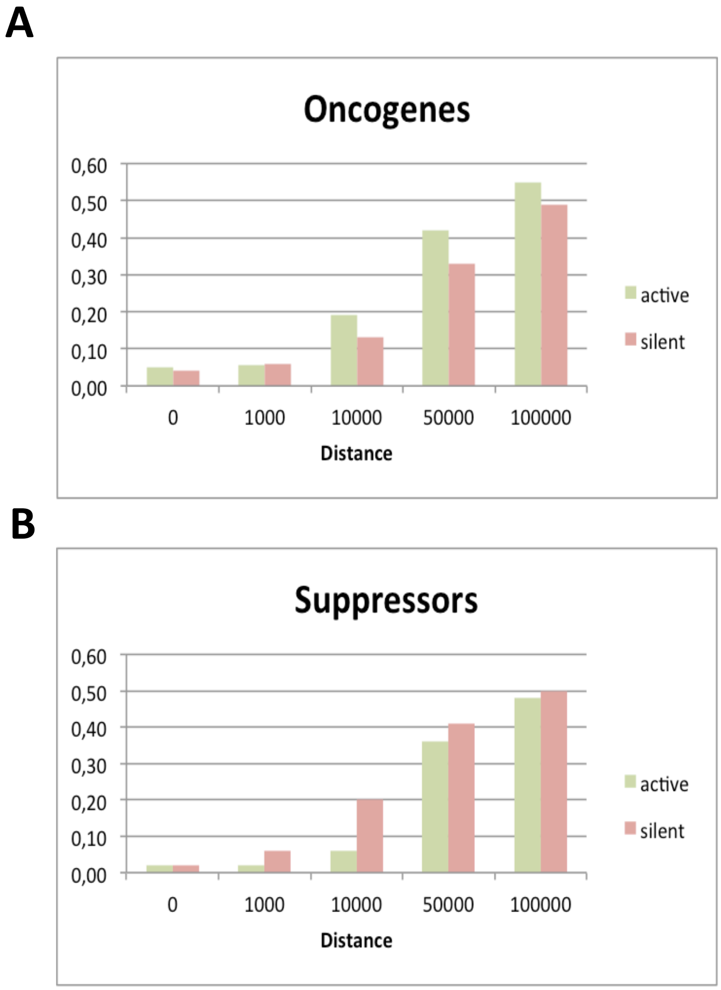

Supplementary Figure 10. These plots show on the y-axis the proportion of the Oncogenes **(A)** or Suppressors **(B)** with an active or silent enhancer at a distance indicated on the x-axis in bp.

Supplementary Figure 11

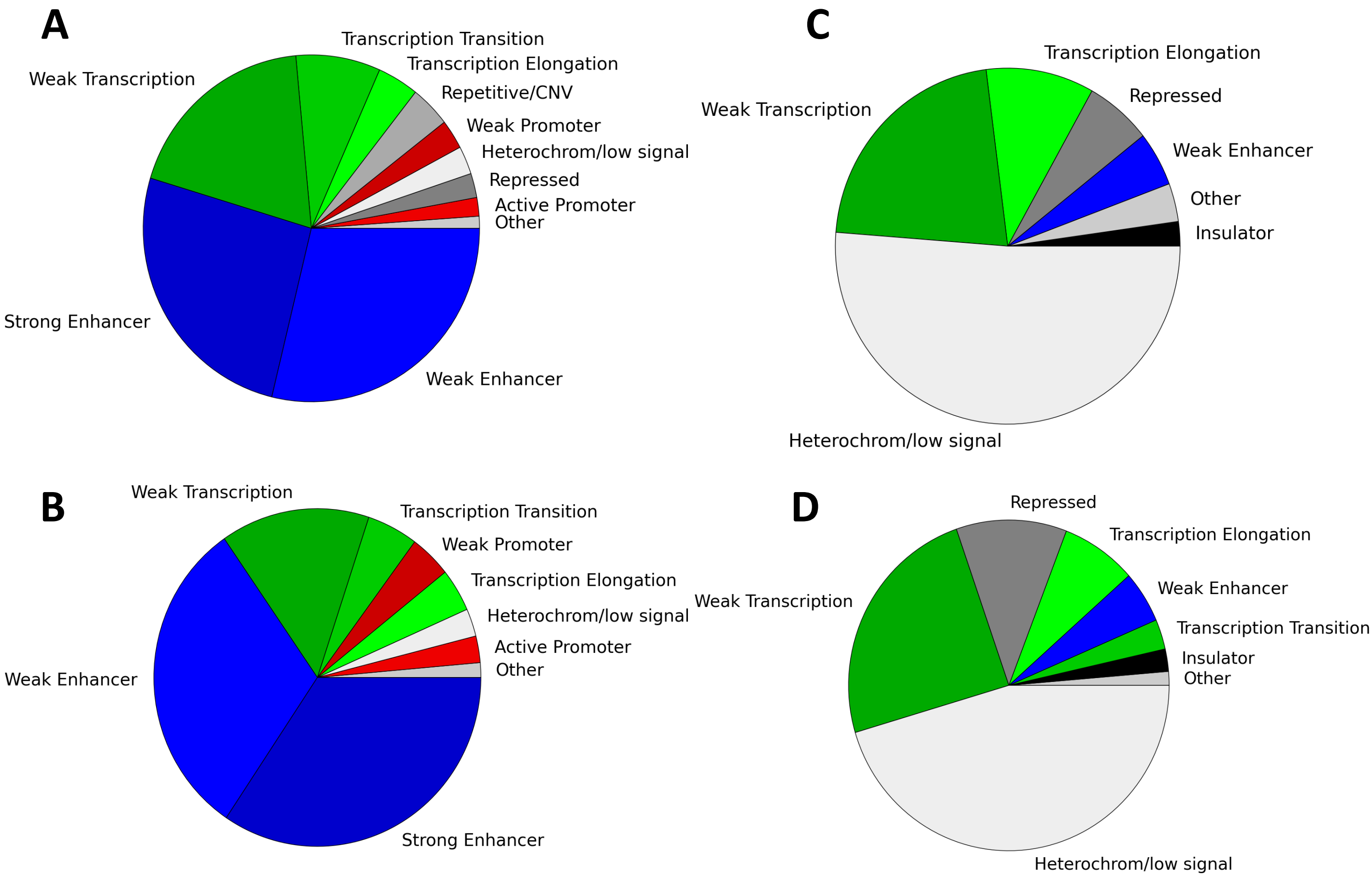

Supplementary Figure 11. Pie charts showing the overlap of **(A)** active intragenic enhancers in K562 with ChromHMM classes for K562, **(B)** active intragenic enhancers in GM12878 with ChromHMM classes for GM12878, **(C)** silent intragenic enhancers in K562 with ChromHMM classes for K562 and **(D)** silent intragenic enhancers in GM12878 with ChromHMM classes for GM12878

Supplementary Figure 11 (Continued)

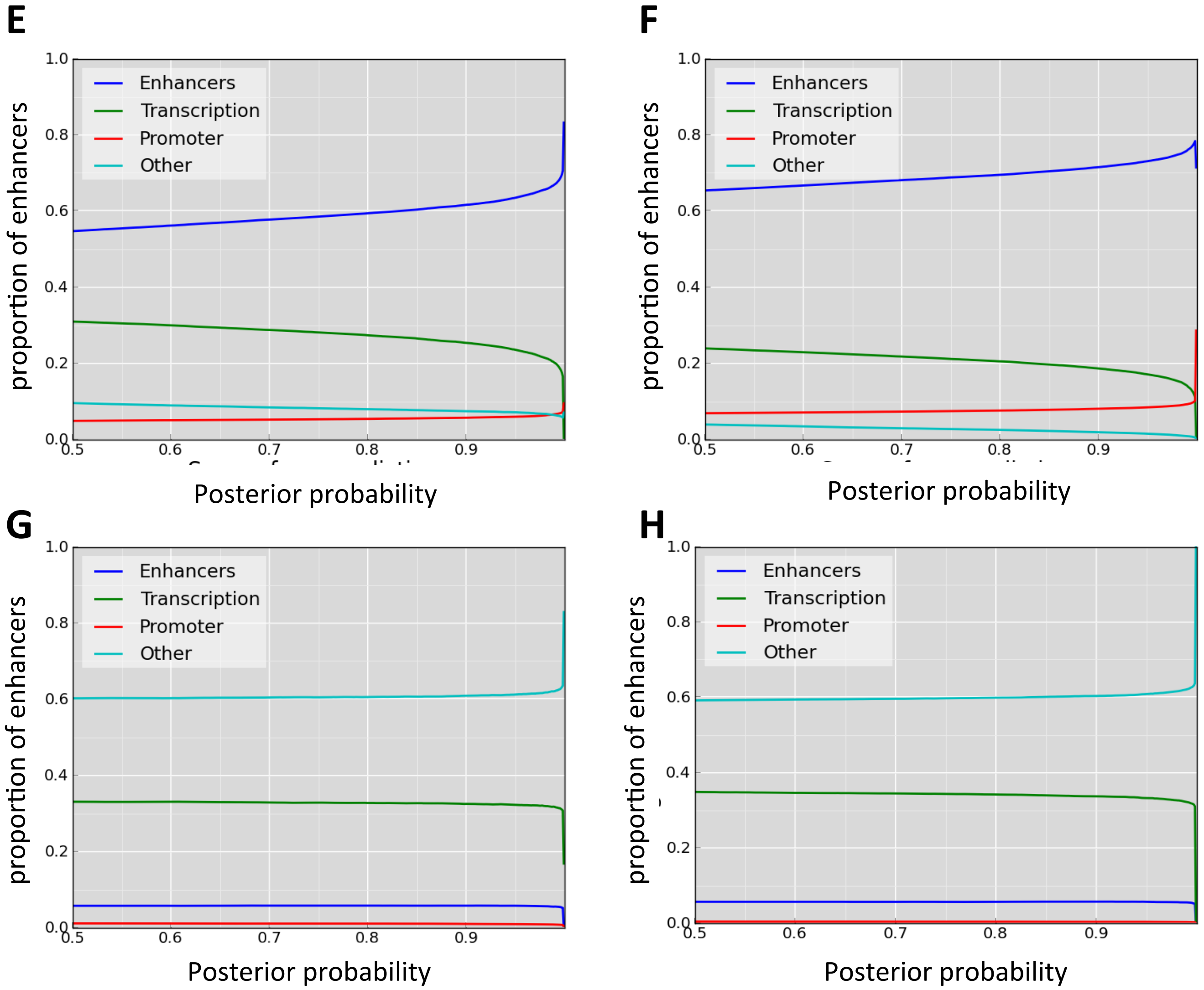

Supplementary Figure 11. Proportion of overlap of predicted enhancers with ChromHMM regions as a function of the posterior probabilities, for active enhancers in K562 **(E)** and GM12878 **(F)** and of silent enhancers in K562 **(G)** and GM12878 **(H),** with regions predicted by ChromHMM in the corresponding cell line. Color codes as in supplementary figure 5.

Supplementary Figure 12

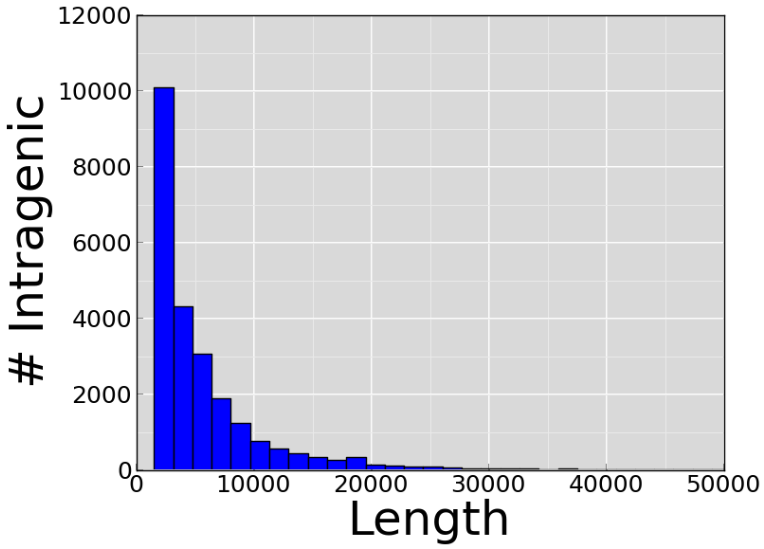

Supplementary Figure 12 - Length distribution of intragenic enhancers

Supplementary Figure 13

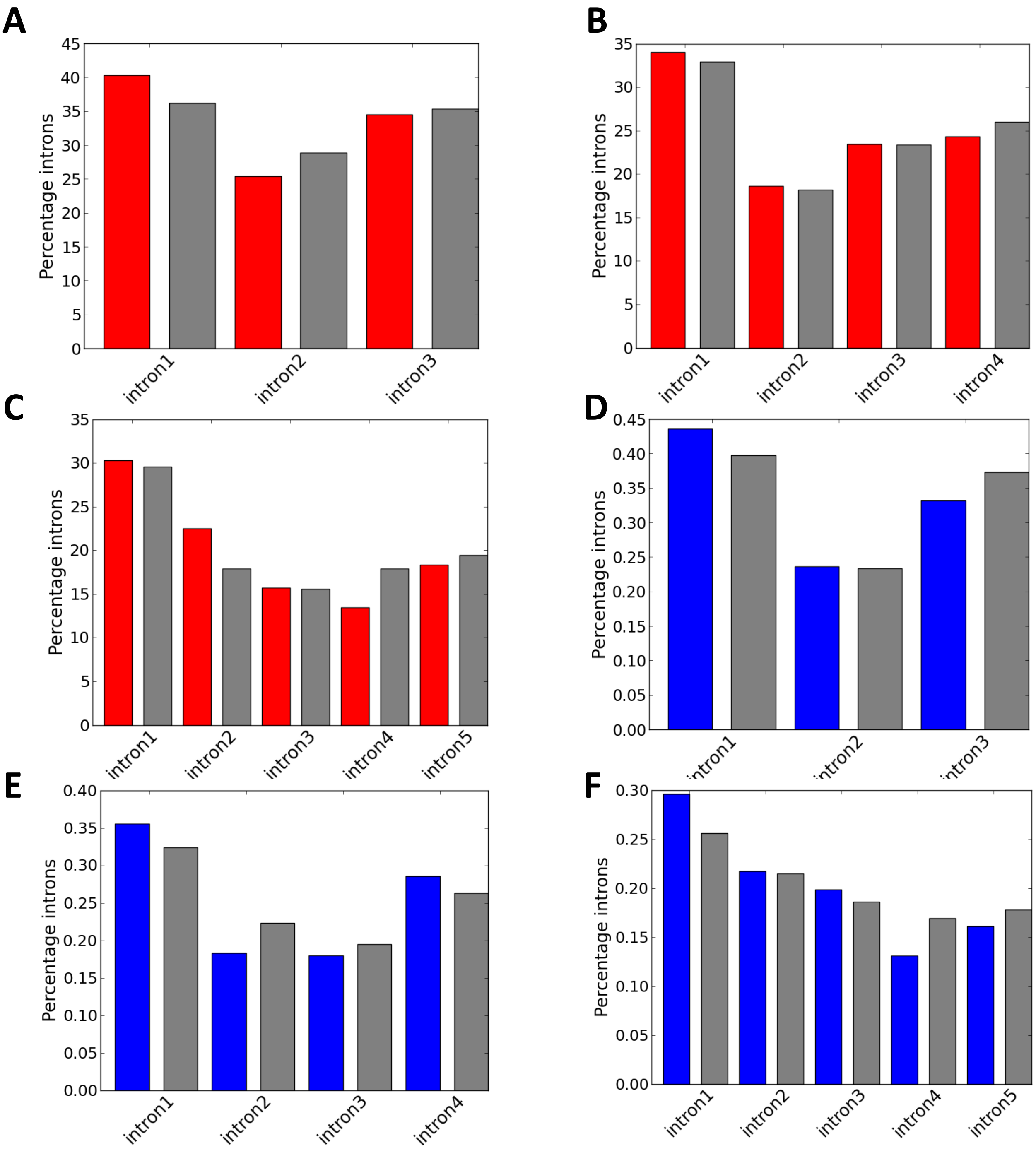

Supplementary Figure 13. These barplots show the positional bias of intronic active and silent enhancers. The y-axes indicate the percentage of enhancer in each intron for active enhancers (red) and randomized enhancers (gray) in genes with just 3 **(A)**, 4 **(B)** or 5 **(C)** introns. Similarly, we show the proportion in each intron of silent enhancers (blue) and randomized enhancers (gray) for genes with just 3 **(D)**, 4 **(E)** or 5 **(F)** introns.

Supplementary Figure 14

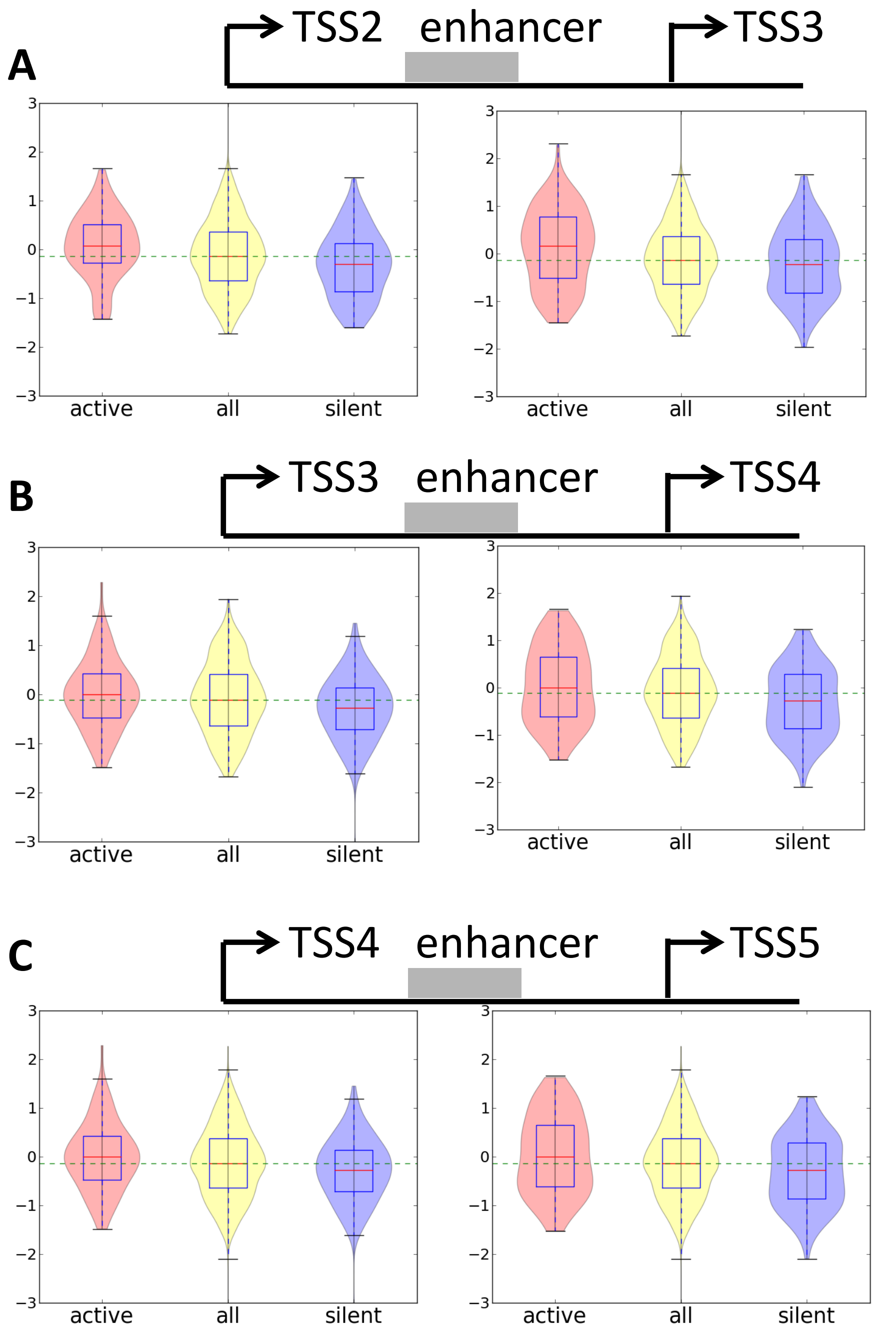

Supplementary Figure 14. RNAPII relative levels at the TSSs flanking intragenic enhancers, for the second and third TSS (A), third and fourth (B) and fourth and fith (C) when they flank an active enhancer (light red), a silent enhancer (light purple) and for the whole set of putative enhancer regions.

Supplementary Figure 15

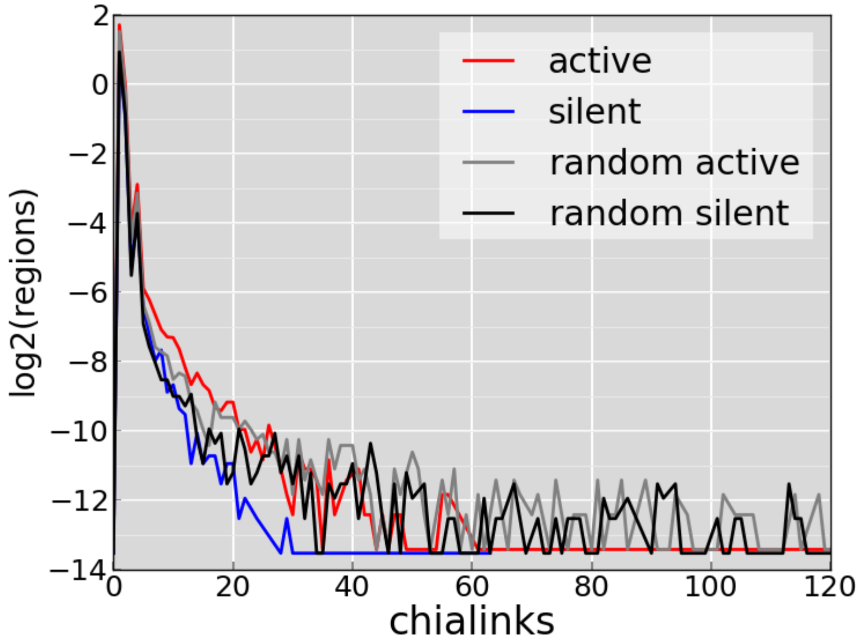

Supplementary Figure 15 - ChIA-PET links for intragenic enhancers. In the Y-axis we plot in log2-scale the fraction of regions with ChIA-PET links to a nearby TSS, for active (red), silent (blue), random active (light grey) and random silent (dark grey) enhancers. TSS – enhancer pairs are considered when all elements are located at least 3 kilobases away and as far as 100 kilobases.

Total genes with 2 intron(s): 5392
Intron 1 avg. length: 7263.61183234
Intron 2 avg. length: 7199.96735905

Total genes with 3 intron(s): 3089
Intron 1 avg. length: 7832.92651343
Intron 2 avg. length: 4888.83360311
Intron 3 avg. length: 6798.17578504

Total genes with 4 intron(s): 2296
Intron 1 avg. length: 7814.87108014
Intron 2 avg. length: 4712.29790941
Intron 3 avg. length: 4521.63719512
Intron 4 avg. length: 6246.63240418

Total genes with 5 intron(s): 1863
Intron 1 avg. length: 6881.82930757
Intron 2 avg. length: 5561.91250671
Intron 3 avg. length: 4552.50187869
Intron 4 avg. length: 4049.90982287
Intron 5 avg. length: 5628.71604938

Total genes with 6 intron(s): 1554
Intron 1 avg. length: 6973.46718147
Intron 2 avg. length: 5309.77284427
Intron 3 avg. length: 4375.55984556
Intron 4 avg. length: 4445.78635779
Intron 5 avg. length: 4006.58687259
Intron 6 avg. length: 4861.94144144

Total genes with 7 intron(s): 1290
Intron 1 avg. length: 6926.83333333
Intron 2 avg. length: 5174.78914729
Intron 3 avg. length: 4665.16744186
Intron 4 avg. length: 4541.41782946
Intron 5 avg. length: 3769.95968992
Intron 6 avg. length: 3817.72868217
Intron 7 avg. length: 4435.38294574

Supplementary Table 1. First introns are longer on average. We calculated the average of every intron separating genes in the GENCODE.V7 annotation by number of introns, from 2 to 7 introns. The only case where the first intron doesn't seem to be longer in the 2 introns genes group.

RNA-Seq	All genes	Genes with regulated events (cytosolic)	Genes with regulated events (nuclear)
Genes with active enhancers in K562	5609	210	229
Genes with no active enhancers in K562	26740	266	294
Genes with active enhancers in GM12878	5905	231	254
Genes with no active enhancers in GM12878	26444	245	269

Supplementary Table 2. Number of genes with and without enhancers active in either cell line that have at least one alternative splicing event that changes between the two cell lines. The regulated events were calculated as described in Methods. Events regulated according to either replicate comparison were considered for the comparison. All genes were multi-exonic.

GO Term	Description	P-value	FDR q-value	Enrichment	N	B	n	b
GO:0044260	cellular macromolecule metabolic process	2.49E-4	1,00E+00	1.31	5363	1928	234	110
GO:0006351	transcription, DNA-templated	2.8E-4	1,00E+00	1.65	5363	653	234	47
GO:0097659	nucleic acid-templated transcription	2.8E-4	9.14E-1	1.65	5363	653	234	47
GO:0010468	regulation of gene expression	5.82E-4	1,00E+00	1.40	5363	1224	234	75
GO:0090304	nucleic acid metabolic process	6.09E-4	1,00E+00	1.44	5363	1084	234	68
GO:0006325	regulation of nucleobase-containing compound metabolic process	7.02E-4	1,00E+00	1.38	5363	1293	234	78
GO:0006325	chromatin organization	7.64E-4	1,00E+00	2.13	5363	226	234	21
GO:0016568	chromatin modification	7.94E-4	9.71E-1	2.17	5363	211	234	20

Supplementary Table 3. Enrichment analysis of the Gene Ontology process for the genes with intragenic enhancers (active or silent in K562) that also contain regulated events ($|\Delta\Psi| > 0.1$ in any of two replicates)

Enhnacers active in GM12878

Cytosolic sample (rep 1)

multi-exonic genes		PSI change	PSI no-change
	with enhancer	166	697
	no enhancer	169	867
	Odds-ratio	1,2217	
	Fisher p-value	0,1027	

Cytosolic sample (rep 2)

multi-exonic genes		PSI change	PSI no-change
	with enhancer	135	589
	no enhancer	155	807
	Odds-ratio	1,19320	
	Fisher p-value	0,1923	

Nuclear sample (rep 1)

multi-exonic genes		PSI change	PSI no-change
	with enhancer	184	633
	no enhancer	181	839
	Odds-ratio	1,3472	
	Fisher p-value	0,01142	

Nuclear sample (rep 2)

multi-exonic genes		PSI change	PSI no-change
	with enhancer	185	586
	no enhancer	193	786
	Odds-ratio	1,2855	
	Fisher p-value	0,0351	

Enhnacers active in K562

Cytosolic sample (rep 1)

multi-exonic genes		PSI change	PSI no-change
	with enhancer	158	643
	no enhancer	177	921
	Odds-ratio	1,2784	
	Fisher p-value	0,04432	

Cytosolic sample (rep 2)

multi-exonic genes		PSI change	PSI no-change
	with enhancer	121	567
	no enhancer	169	829
	Odds-ratio	1,0468	
	Fisher p-value	0,7429	

Nuclear sample (rep 1)

multi-exonic genes		PSI change	PSI no-change
	with enhancer	174	593
	no enhancer	191	879
	Odds-ratio	1,3478	
	Fisher p-value	0,01272	

Nuclear sample (rep 2)

multi-exonic genes		PSI change	PSI no-change
	with enhancer	160	550
	no enhancer	218	822
	Odds-ratio	1,0969	
	Fisher p-value	0,4423	

Supplementary Table 4. Contingency tables with the gene counts with or without active enhancers in GM12878 (left) or in K562 (right), separated according to whether they have alternative splicing events that change the PSI (|delta PSI|>0.1) or not (| delta PSI| < 0.05). The delta PSI calculation was performed twice, one for each pairing of one replicate from each cell line. Other pairings did not produce any mayor changes in the results. All odds-ratios are > 1. Fisher p-values < 0.05 are marked in green

Enhancers active in GM12878

Cytosolic sample (rep 1)

multi-exonic genes, no diff-exp		PSI change	PSI no-change
	with enhancer	124	497
	no enhancer	109	642
	Odds-ratio	1,4691	
	Fisher p-value	0,009215	

Cytosolic sample (rep 2)

multi-exonic genes, no diff-exp		PSI change	PSI no-change
	with enhancer	97	432
	no enhancer	106	599
	Odds-ratio	1,2680	
	Fisher p-value	0,1403	

Nuclear sample (rep 1)

multi-exonic genes, no diff-exp		PSI change	PSI no-change
	with enhancer	127	463
	no enhancer	123	609
	Odds-ratio	1,3578	
	Fisher p-value	0,03394	

Nuclear sample (rep 2)

multi-exonic genes, no diff-exp		PSI change	PSI no-change
	with enhancer	137	425
	no enhancer	126	558
	Odds-ratio	1,4271	
	Fisher p-value	0,01196	

Enhancers active in K562

Cytosolic sample (rep 1)

multi-exonic genes, no diff-exp		PSI change	PSI no-change
	with enhancer	109	464
	no enhancer	124	675
	Odds-ratio	1,2785	
	Fisher p-value	0,09378	

Cytosolic sample (rep 2)

multi-exonic genes, no diff-exp		PSI change	PSI no-change
	with enhancer	93	420
	no enhancer	110	611
	Odds-ratio	1,22972	
	Fisher p-value	0,186	

Nuclear sample (rep 1)

multi-exonic genes, no diff-exp		PSI change	PSI no-change
	with enhancer	124	419
	no enhancer	126	653
	Odds-ratio	1,5332	
	Fisher p-value	0,002691	

Nuclear sample (rep 2)

multi-exonic genes, no diff-exp		PSI change	PSI no-change
	with enhancer	117	392
	no enhancer	146	591
	Odds-ratio	1,2080	
	Fisher p-value	0,1804	

Supplementary Table 5. The same as Supplementary Table 3, but the genes considered are those that do change expression between the two cell lines (Methods). All odds-ratios are > 1. Fisher p-values < 0.05 are marked in green

Supplementary Figure 16

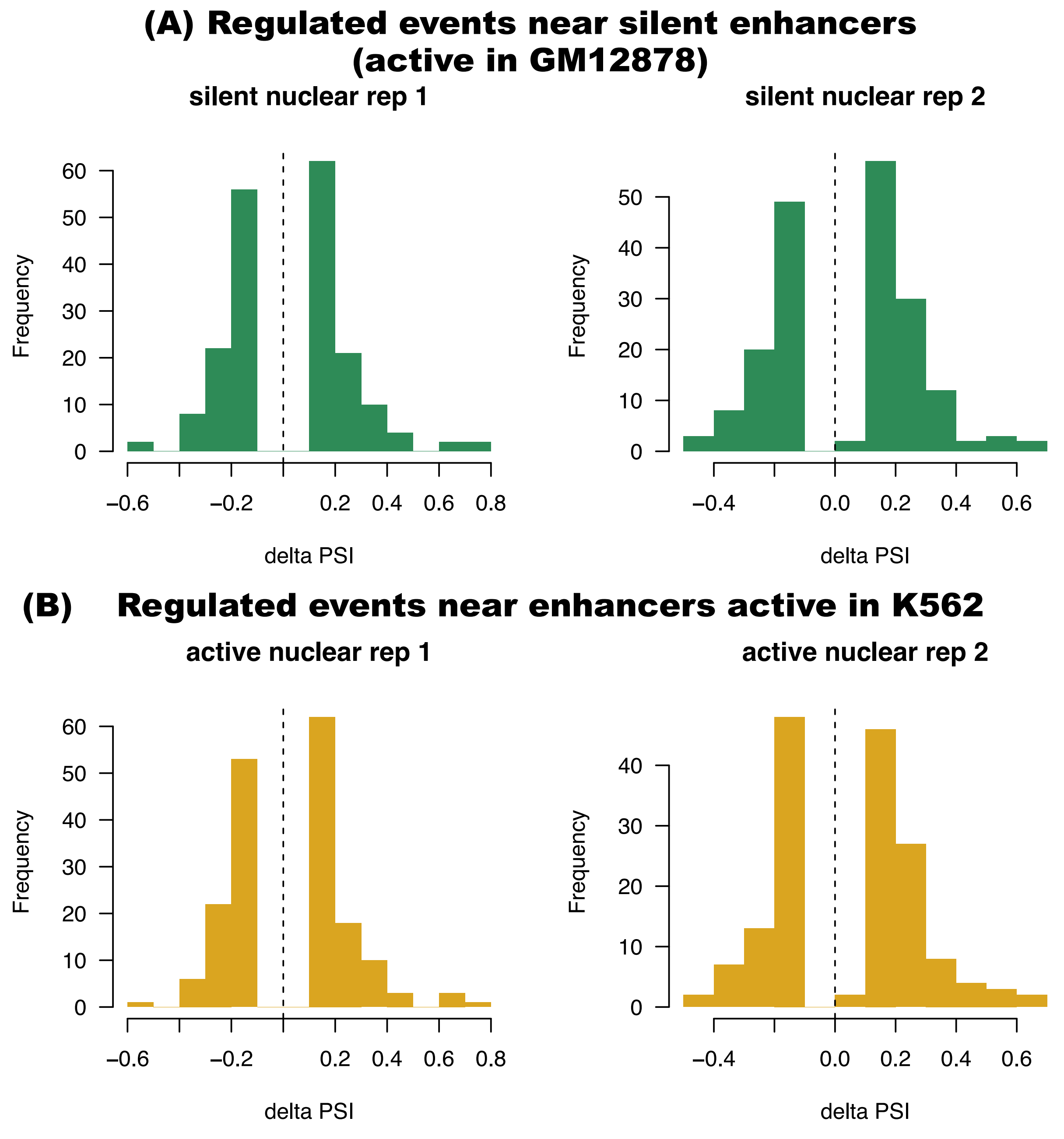

Supplementary Figure 16. Distributions of the delta PSI values of the events regulated nearby active enhancers. Regulated events were calculated as those exon cassettes that had |delta PSI| > 0.1 comparing the PSI in K562 and GM12878. Only the closest enhancer is selected for each event. Two comparisons were made, matching each replicate in one cell line with one replicate in the other cell line.

Supplementary Figure 17

(A) Regulated events near silent enhancers (active in GM12878)

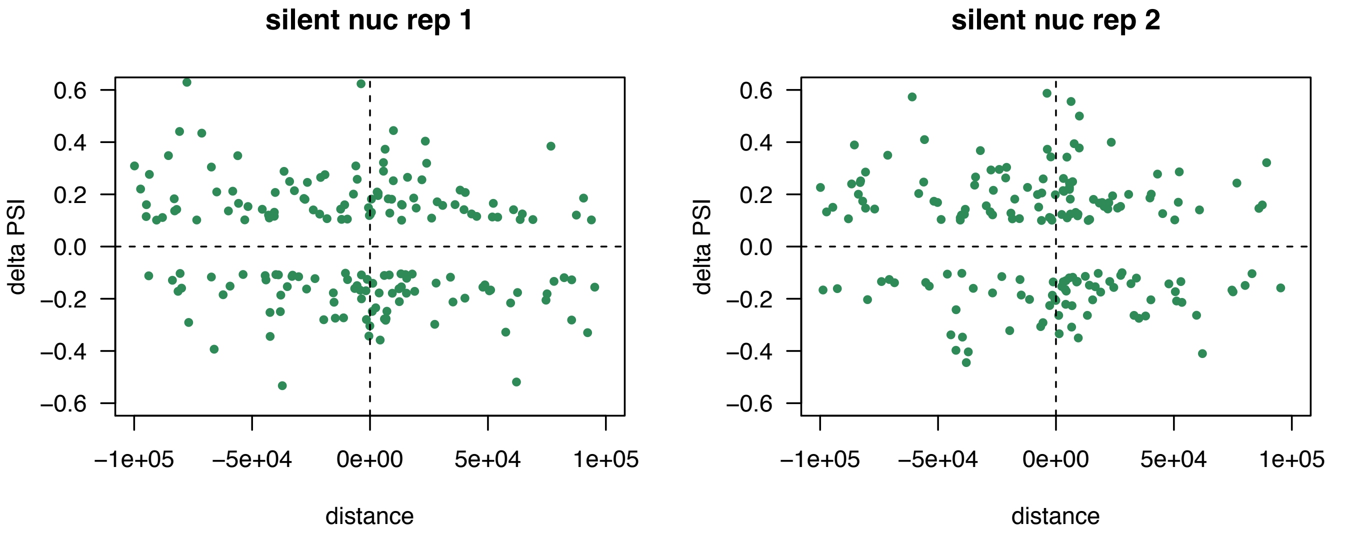

(B) Regulated events near enhancers active in K562

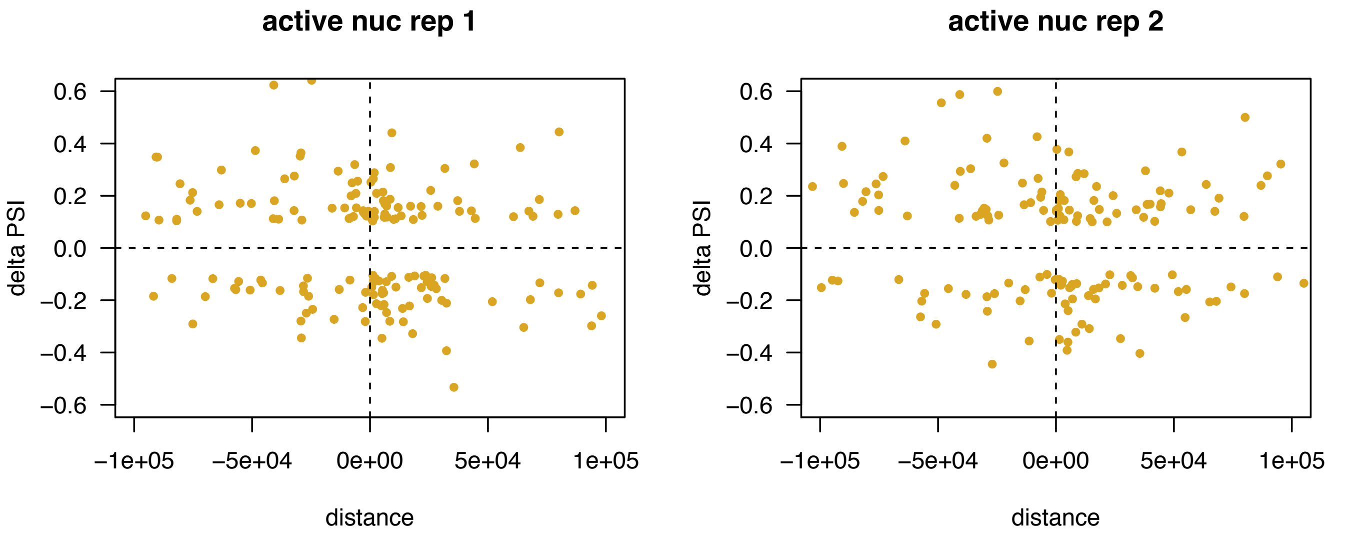

Supplementary Figure 17. Relation between the delta PSI values (y axis), calculated as the difference of PSI between K562 and GM12878, and the distance to the closest enhancer in nucleotides (x axis). Negative and positive distances correspond to upstream and downstream positions, respectively. The distance is calculated from the middle point of the regulated exon to the middle point of the predicted enhancer. The plots show the delta PSI values calculated for the two replicate comparisons (left and right panels) for each set of enhancers, active in GM12878 (silent in K562) **(A)** or active in K562 **(B)**.

Supplementary Figure 18

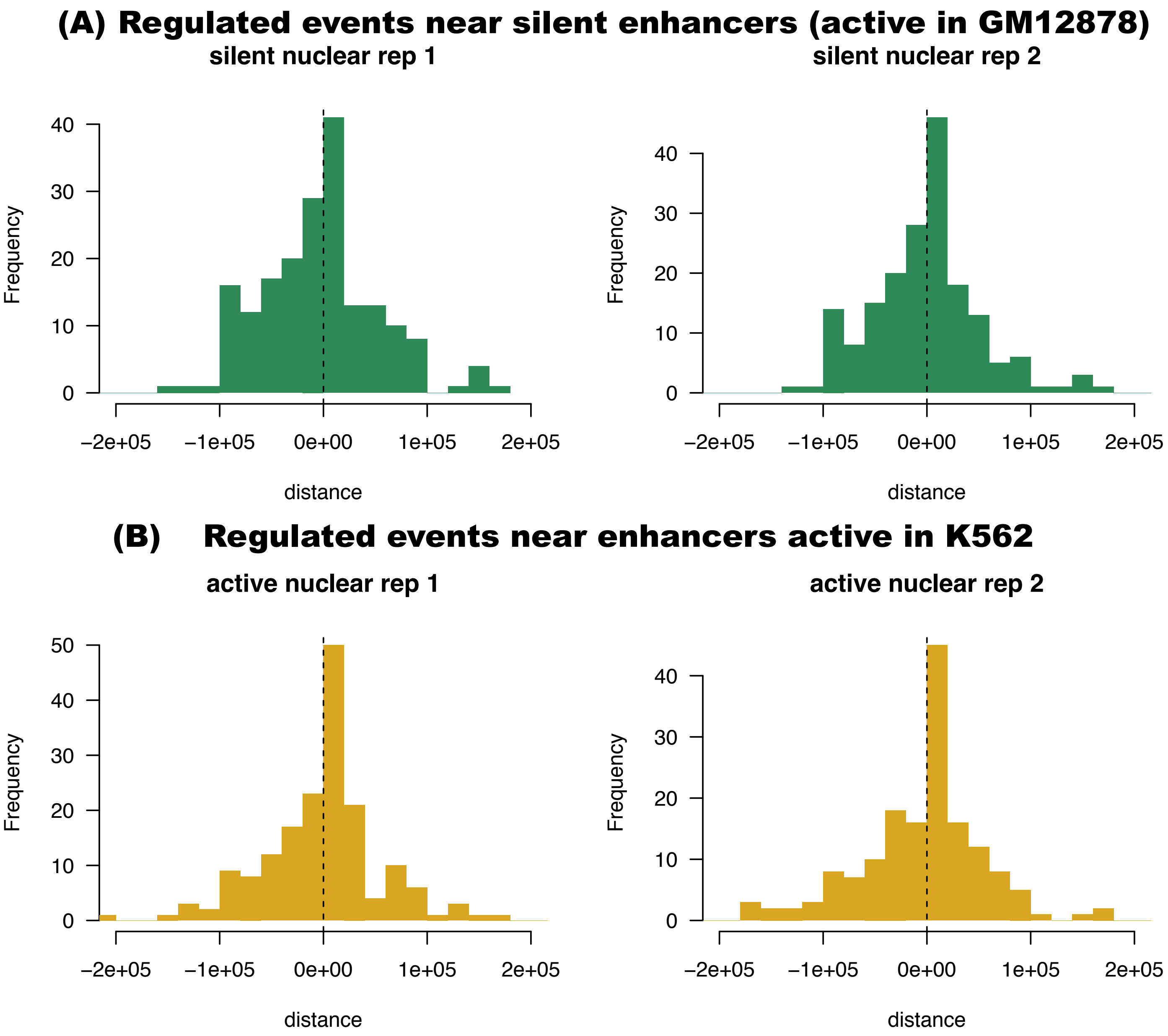

Supplementary Figure 18. Distribution of the distances between the regulated events and the closest enhancer shown in Supplementary Figure 16. Negative and positive distances correspond to upstream and downstream positions, respectively. The distance is calculated from the middle point of the regulated exon to the middle point of the predicted enhancer. The plots show distributions for the two replicates (left and right panels) for each set of enhancers, active in GM12878 (silent in K562) **(A)** or active in K562 **(B)**.

Supplementary Figure 19

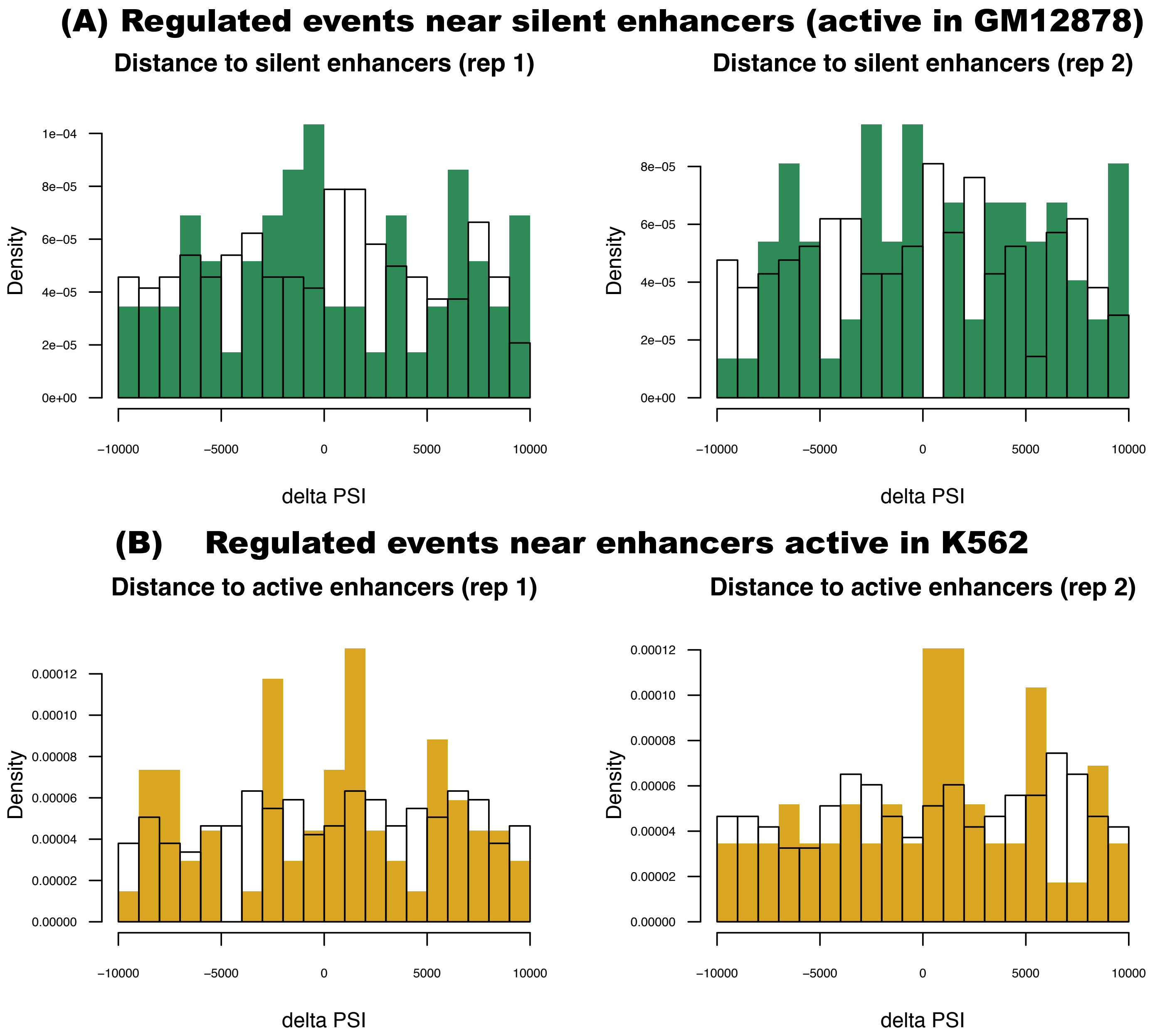

Supplementary Figure 19. Distribution of the distances to the closest enhancer for regulated (colored bars) and non-regulated (empty bars) events. Only events genes with only regulated or non-regulated events were considered for this comparison. Negative and positive distances correspond to upstream and downstream positions, respectively. The distance is calculated from the middle point of the regulated exon to the middle point of the predicted enhancer. The plots show distributions for the two replicates (left and right panels) for each set of enhancers, active in GM12878 (silent in K562) **(A)** or active in K562 **(B)**.
